# Psychometric Properties of the Japanese Version of the Motor Activity Log in Patients With Upper Extremity Orthopedic Disorders

**DOI:** 10.7759/cureus.106487

**Published:** 2026-04-05

**Authors:** Kaoru Sasagawa, Wataru Kukizaki, Naoki Ohkusa, Kayoko Takahashi, Ryota Hayasaki

**Affiliations:** 1 Department of Rehabilitation, Medical Base Shinkoiwa, Tokyo, JPN; 2 Division of Occupational Therapy, Department of Rehabilitation, Faculty of Health Science, Kumamoto Health Science University, Kumamoto, JPN; 3 Department of Rehabilitation, Kenwakai Otemachi Hospital, Kitakyushu, JPN; 4 Occupational Therapy Course, Department of Rehabilitation, School of Allied Health Science, Kitasato University, Kanagawa, JPN; 5 Graduate School of Medical Sciences, Kitasato University, Kanagawa, JPN; 6 Department of Occupational Therapy, School of Health Sciences, Sapporo Medical University, Sapporo, JPN

**Keywords:** injury, motor activity log, orthopedic disorders, performance, quickdash

## Abstract

Introduction: Evaluating real-world arm use ("performance") is crucial in orthopedic rehabilitation; however, existing tools often measure only subjective difficulty. The Motor Activity Log (MAL) assesses real-world use but has not been validated in orthopedic populations. This study was conducted to examine the reliability and validity of the Japanese version of the MAL in patients with upper extremity orthopedic disorders. Materials and methods: In this cross-sectional study, 30 patients with upper extremity orthopedic disorders completed the Japanese version of the MAL, which comprises the amount of use (AOU) and quality of movement (QOM) scales. Reliability was assessed using Cronbach’s α for internal consistency, intraclass correlation coefficients (ICC) for test-retest reliability, and Bland-Altman analysis for systematic error. Construct validity was evaluated using Spearman’s rank correlation coefficients to analyze relationships with the QuickDASH (Quick Disabilities of the Arm, Shoulder, and Hand), the Visual Analog Scale (VAS) for pain, and grip strength. Results: Cronbach’s α was 0.96 for both scales of the MAL, indicating high internal consistency. Test-retest reliability was excellent (ICC > 0.99). Bland-Altman analysis revealed no fixed or proportional bias. Regarding validity, the results supported the hypothesized pattern of strong convergent validity with the QuickDASH (r = -0.87 to -0.83) and VAS (r = -0.64 to -0.51). Discriminant validity was supported by moderate correlations with grip strength (r = 0.50), indicating that the MAL captures a construct distinct from physical capacity.

Conclusions: Our findings support the view that the Japanese version of the MAL has high reliability and validity for assessing real-world arm use in patients with orthopedic disorders. It effectively measures "performance," which is distinct from "capacity" or "subjective difficulty." This tool is clinically useful for identifying discrepancies between functional recovery and actual use, particularly in the presence of pain-induced protective behavior. Combining the MAL with existing tools, such as the QuickDASH, allows clinicians to evaluate whether functional recovery translates into real-world arm use, facilitating targeted rehabilitation strategies.

## Introduction

Research on traumatic hand injuries indicates that many patients continue to experience impacts on daily living and social participation even after completing rehabilitation [1]. Furthermore, moderate-to-severe difficulty is reported more frequently in the work (40.8%) and leisure (46.0%) domains than in the self-care (28.0%) domain, indicating a widespread impact [1]. Currently, functional assessment of upper extremity orthopedic disorders widely uses the Disabilities of the Arm, Shoulder, and Hand (DASH) [2] as a general subjective measure, along with disease-specific scales such as the American Shoulder and Elbow Surgeons (ASES) score and the Patient-Rated Wrist Evaluation (PRWE). While these measures are useful for comprehensively capturing pain, range of motion (ROM), muscle strength, and perceived difficulty, they reflect patients' self-perceived functional limitations or symptom severity. However, they do not directly evaluate "performance," which refers to whether the affected limb is actually used in real-world situations. Considering that the ultimate goal of upper extremity orthopedic rehabilitation is not merely functional recovery but the promotion of actual use of the affected limb in daily life, it is essential to introduce a new metric that can quantify not only "capacity" (what one can do) but also "performance" (what one actually does).

In rehabilitation science, it is crucial to conceptually distinguish between "capacity" and "performance." Capacity refers to the highest level of functioning an individual can achieve in a standardized environment. In orthopedic practice, this is typically measured not only through patient-reported outcome measures but also through clinician-rated and device-based assessments, such as grip strength measurements with dynamometers and range-of-motion tests with goniometers. Conversely, performance refers to what patients actually do in their real-world environment. In orthopedic settings, a significant gap often exists between these two constructs, primarily driven by pain-related fear or protective behavior [3]. Since the ultimate goal of upper extremity orthopedic rehabilitation is not merely functional recovery (improvement in capacity) but the actual use of the affected limb in daily life (performance improvement), it is essential to implement new indicators that quantify performance in addition to capacity.

In stroke rehabilitation, the Motor Activity Log (MAL) is widely used as a semi-structured interview-based scale to evaluate real-world upper extremity use. The MAL assesses the amount of use (AOU) and quality of movement (QOM) of the affected limb across 14 important daily activities. Uswatte et al. [4] reported that the MAL captures real-world usage frequency with high reliability, making it an indispensable tool for assessing outcomes in interventions such as constraint-induced movement therapy. The Japanese version of the MAL was developed by Takahashi et al. [5] following a rigorous translation process based on 36-Item Short Form Health Survey protocols. Specifically, forward and backward translations were performed, and consistency between the original and back-translated versions was rated on an 11-point scale (0-10) to ensure linguistic and conceptual equivalence through repeated revisions. Its clinical utility has been verified in patients with hemiparetic stroke, demonstrating extremely high internal consistency (Cronbach’s α: AOU = 0.97, QOM = 0.98) and established criterion-related validity, as evidenced by significant correlations with the Brunnstrom stage and the five-task test (r = 0.64-0.81) [5]. Thus, while the reliability and validity of the Japanese MAL are well established in the cerebrovascular domain, its applicability to musculoskeletal disorders remains unverified.

When applying the MAL to musculoskeletal disorders, it is critical to articulate how the mechanism of arm nonuse differs from that in stroke populations. In neurological conditions, "learned nonuse" primarily stems from primary sensorimotor deficits, in which repeated failed attempts to use the paretic limb lead to behavioral suppression and compensatory use of the unaffected limb [6]. Conversely, nonuse in orthopedic disorders is hypothesized to follow a different pathway. According to a model proposed by Punt et al. [7], injury-induced pain and enforced immobility lead to movement suppression and fear-avoidant behavior, which eventually establishes "learned nonuse" independent of central nervous system lesions. Based on this theoretical framework, we formulated an a priori hypothesis that nonuse among patients with upper extremity orthopedic disorders is predominantly "protective nonuse." Consequently, we hypothesized that MAL scores in this orthopedic population would show stronger correlations with pain intensity (a primary driver of fear-avoidance) than with purely mechanical capacity, such as grip strength.

Therefore, the purpose of this study was to examine the reliability and validity of the Japanese version of the MAL in patients with upper extremity orthopedic disorders. Specifically, the aim was to verify the internal consistency and test-retest reliability of the MAL and to clarify its construct validity (convergent and discriminant) by analyzing its associations with existing measures, such as the QuickDASH (Quick Disabilities of the Arm, Shoulder, and Hand) and pain scales.

## Materials and methods

Study design and participants

This cross-sectional study was conducted in December 2025. Participants were 30 patients with upper extremity orthopedic disorders who visited the outpatient rehabilitation department at a general hospital in Japan. Participants were recruited via convenience sampling from eligible patients who visited our clinic during the study period.

The inclusion criteria were the following: (1) diagnosis of an upper extremity musculoskeletal disorder by a physician and (2) the ability to understand instructions in Japanese. The exclusion criteria were the following: (1) history of central nervous system diseases, (2) difficulty responding to questionnaires due to cognitive decline, and (3) severe pain that makes physical function tests difficult to perform. This exclusion was methodologically necessary because including patients with extreme pain-induced nonuse would cause a profound floor effect on the MAL scores, hindering the adequate evaluation of the scale's psychometric properties.

The Institutional Review Board of Kenwakai Otemachi Hospital approved the study design (approval number: 25018). All participants received verbal and written explanations of the study's purpose and methods and provided written informed consent.

Sample size calculation

Sample size was calculated using G*Power software (version 3.1.9.7; Heinrich Heine University Düsseldorf, Düsseldorf, Germany) [8]. For correlation analysis, which was the primary objective of this study, the required sample size was calculated to be 26 participants, based on a statistical power (1-β) of 0.80, a significance level (α) of 0.05, and an effect size (correlation coefficient p) of 0.50 (strong correlation). This effect size was selected because previous validation studies of the MAL have reported correlation coefficients of 0.50 or higher between the MAL and other functional assessments [5]. The 30 participants included in this study met this criterion, indicating sufficient statistical power.

Clinical assessments

Basic attributes, including age, sex, dominant hand, diagnosis, and disease duration, were obtained from medical records.

Japanese MAL

We used a modified Japanese version of the MAL for patients with upper extremity orthopedic disorders [5]. Regarding the modified Japanese MAL, its developer, Dr. Kayoko Takahashi, is a co-author of this study. Therefore, its modification and use were conducted with her direct approval. The modification was limited to the adjustment of terminology (e.g., changing 'before the onset of stroke' to 'before the onset of upper extremity orthopedic disorders') to ensure relevance to the target population. The MAL is a semi-structured interview scale in which patients rate 14 daily activities on a six-point scale ranging from 0 (never used/unable to use) to 5 (same as pre-injury/normal) regarding "AOU" and "QOM." The score was calculated as the mean of the items. Consistent with the standard administration of the MAL, the AOU and QOM scores were calculated independently, and a composite score was not calculated.

QuickDASH

The Japanese version of the QuickDASH was used as a patient-reported outcome measure of upper extremity function and symptoms [9]. The QuickDASH is a widely recognized open-access tool. It consists of 11 items assessing difficulty in daily activities and symptoms, rated from 0 (no difficulty) to 5 (unable). The score is converted to a range of 0 (no disability) to 100 (most severe disability). Higher scores indicate more severe functional impairment in daily life.

Grip Strength

Grip strength on the affected side was measured using a Smedley-type hand dynamometer (Sakai Medical Co., Ltd., Tokyo, Japan). Measurements were taken in either a standing or sitting position with the elbow extended. The maximum value of two trials was used.

Pain

Pain intensity at the affected site was measured using a Visual Analog Scale (VAS) [10]. The VAS is a widely recognized open-access tool. Current pain (or pain during motion) was measured on a 100-mm line anchored by "no pain" (0 mm) and "worst imaginable pain" (100 mm).

Procedure

The MAL was administered as a semi-structured interview by a single occupational therapist trained in accordance with the original manual. At the initial assessment, the MAL, QuickDASH, VAS, functional independence measure (FIM), and grip strength were measured on the same day. To examine test-retest reliability, the same examiner readministered the MAL within one to 12 days after the initial assessment. Only cases with no significant changes in pain or functional status and judged to be clinically stable were included in the retest analysis. Specifically, "clinically stable" was defined by the following three criteria: (1) no new injuries, (2) no changes in medical or rehabilitation treatments, and (3) the patient's subjective report of no symptom exacerbation.

Statistical analysis

Statistical analyses were performed using EZR version 1.53 (Saitama Medical Center, Jichi Medical University, Saitama, Japan) [11]. The significance level was set at 5% (two-tailed).

Reliability

Internal consistency was assessed by calculating Cronbach’s α coefficients for AOU and QOM, with values of 0.70 or higher considered sufficient [12]. Test-retest reliability (intra-rater reliability) was examined using the intraclass correlation coefficient (ICC 1,1) and its 95% confidence interval (CI) [13]. Based on previous research [14], ICC values were interpreted as follows: moderate (0.50-0.75), good (0.75-0.90), and excellent (>0.90).

The standard error of measurement (SEM) and minimal detectable change (MDC) were calculated as indices of measurement error using the following formulas [15]: $SEM = SD \cdot \sqrt{1 - ICC}$ and $MDC = SEM \cdot 1.96 \cdot \sqrt{2}$.

Bland-Altman analysis was performed to statistically clarify the presence of systematic errors [16]. A plot was created with the difference between the two measurements on the y-axis and the mean on the x-axis. If the 95% CI of the mean difference did not include 0, a fixed bias was judged to be present [17]. A significant correlation between the difference and the mean was judged to indicate proportional bias. The limits of agreement (LOA) were defined as the mean difference ± 1.96 × standard deviation (SD) of the difference. The 95% CI for the upper and lower limits of the LOA was estimated using the following formula: $LOA\ 95\%\ CI = (Mean \pm 1.96 \times SD) \pm t \times \sqrt{3SD^2/n}$ where (Mean) is the mean of the differences, (SD) is the standard deviation of the differences, (t) is the t-value corresponding to a two-sided P = 0.05, and (n) is the sample size.

Validity

Construct validity was examined by computing Spearman's rank correlation coefficients between the MAL and the QuickDASH (disability/symptom), QuickDASH (work), VAS, FIM, and grip strength (affected and unaffected sides). Based on previous research [18], correlation strength was defined as follows: weak (0.10-0.39), moderate (0.40-0.69), strong (0.70-0.89), and very strong (>0.90).

## Results

Participant characteristics

This study included 30 participants, comprising 13 men (40.0%) and 17 women (60.0%), with an average age of 57.5 ± 17.7 years (range: 20-83 years). The affected side was the right in 20 patients (66.7%) and the left in 10 (33.3%), with dominant hand involvement in 20 patients (67.0%).

Regarding diagnosis, fractures were the most common (n = 22, 73.3%), including humerus fractures (n = 9, 30.0%) and distal radius/wrist fractures (n = 5, 16.7%). Other orthopedic conditions included tendon/tenosynovial disorders (n = 3, 10.0%) and nerve disorders or amputations (n = 2, 6.7% each) (Table 1).

**Table 1:** Participant characteristics (n = 30) SD: standard deviation, N/A: not applicable

Characteristics	No. of patients (%)	Mean (SD)	Range
Age (years)	N/A	57.5 (17.7)	20-83
Sex	N/A	N/A	N/A
Men	13 (40)	N/A	N/A
Women	17 (60)	N/A	N/A
Dominant hand	N/A	N/A	N/A
Right hand	20 (66.7)	N/A	N/A
Left hand	10 (33.3)	N/A	N/A
Dominant hand concordance	20 (67)	N/A	N/A
Diagnoses	N/A	N/A	N/A
Fracture	N/A	N/A	N/A
Shoulder/clavicle	2 (6.7)	N/A	N/A
Humerus	9 (30)	N/A	N/A
Elbow/proximal forearm	3 (10)	N/A	N/A
Distal forearm/wrist	5 (16.7)	N/A	N/A
Hand/fingers	3 (10)	N/A	N/A
Nerve disorders	2 (6.7)	N/A	N/A
Tendon and tenosynovial disorders	3 (10)	N/A	N/A
Amputations	2 (6.7)	N/A	N/A
Joint disorders	1 (3.3)	N/A	N/A

Descriptive statistics

As shown in Table 2, the mean MAL score was 3.5 ± 1.3 for AOU and 3.4 ± 1.3 for QOM, with no missing item responses, as the scale was administered via semi-structured interviews. The mean VAS (pain during motion) score was 41 ± 23 mm, the FIM total score was 122.7 ± 10.4, unaffected grip strength was 25.1 ± 10.4 kg, affected grip strength was 16.1 ± 10.3 kg, and the QuickDASH score was 29.8 ± 20.4.

**Table 2:** Absolute values of all scores at the first test (n = 30) SD: standard deviation, CI: confidence interval, N/A: not applicable, MAL(AOU): Motor Activity Log (amount of use), MAL(QOM): Motor Activity Log (quality of movement), Q-DASH: Quick-Disabilities of the Arm, Shoulder, and Hand questionnaire, VAS: Visual Analog Scale, FIM: functional independence measure

Variables	Mean (SD)	Range	95% CI
MAL(AOU) average score	3.5 (1.3)	0.5-5	3.0-3.9
MAL(QOM) average score	3.4 (1.3)	0.4-5	3.0-3.9
Q-DASH	N/A	N/A	N/A
Disability/symptom	29.8 (20.4)	0-70.5	22.2-37.4
VAS (mm)	41 (23)	5-74	32.1-49.3
FIM	122.7 (10.4)	106-126	120.4-124.9
Grip strength (kg)	N/A	N/A	N/A
Affected side	16.1 (10.3)	0-41.1	12.2-20.0
Unaffected side	25.1 (10.4)	10.4-40.2	21.2-29.0

Reliability

Cronbach’s α coefficient for assessing internal consistency was 0.96 for AOU and 0.96 for QOM, indicating very high consistency (Table 3).

**Table 3:** Internal consistency and construct validity of the MAL (n = 30) MAL(AOU): Motor Activity Log (amount of use), MAL(QOM): Motor Activity Log (quality of movement), Q-DASH: Quick Disabilities of the Arm, Shoulder, and Hand questionnaire, VAS: Visual Analog Scale, FIM: functional independence measure, N/A: not applicable, r: Spearman’s rank correlation coefficient * indicates p < 0.05

Variables	Cronbach’s α	MAL(AOU) (r)	MAL(QOM) (r)	Q-DASH (r)	VAS (r)	FIM (r)	Grip strength (affected-side) (r)	Grip strength (unaffected-side) (r)
MAL(AOU)	0.96	N/A	0.90*	−0.87*	−0.64*	0.51*	0.50*	0.29
MAL(QOM)	0.96	0.90*	N/A	−0.83*	−0.51*	0.43*	0.50*	0.32

**Figure 1:**
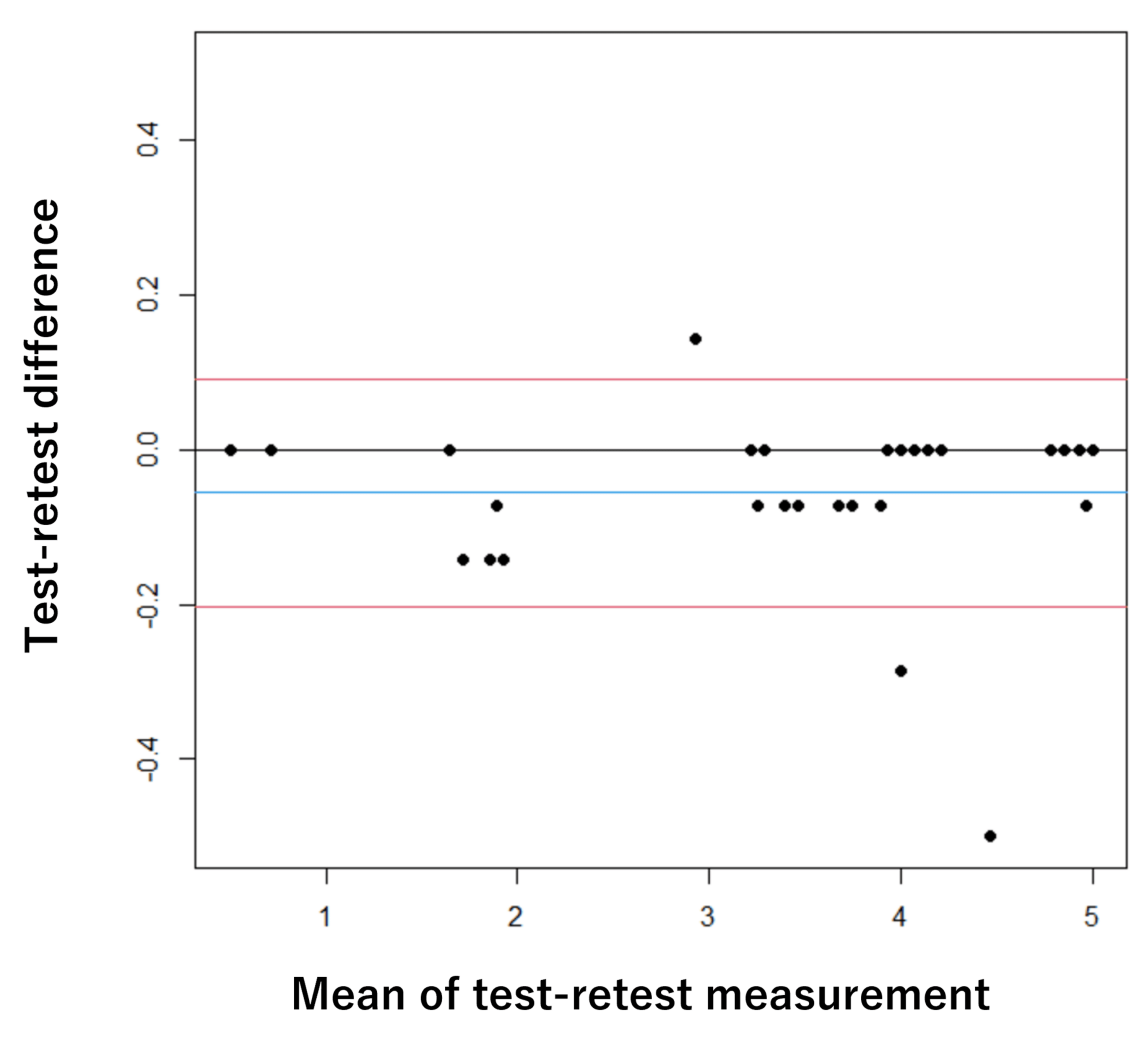
Bland-Altman plot describing the test–retest reliability of MAL(AOU) The blue line indicates the mean, the grey line indicates zero, and the red lines mark the lower and upper bounds of the 95% CI. MAL(AOU): Motor Activity Log (amount of use), CI: confidence interval

**Figure 2:**
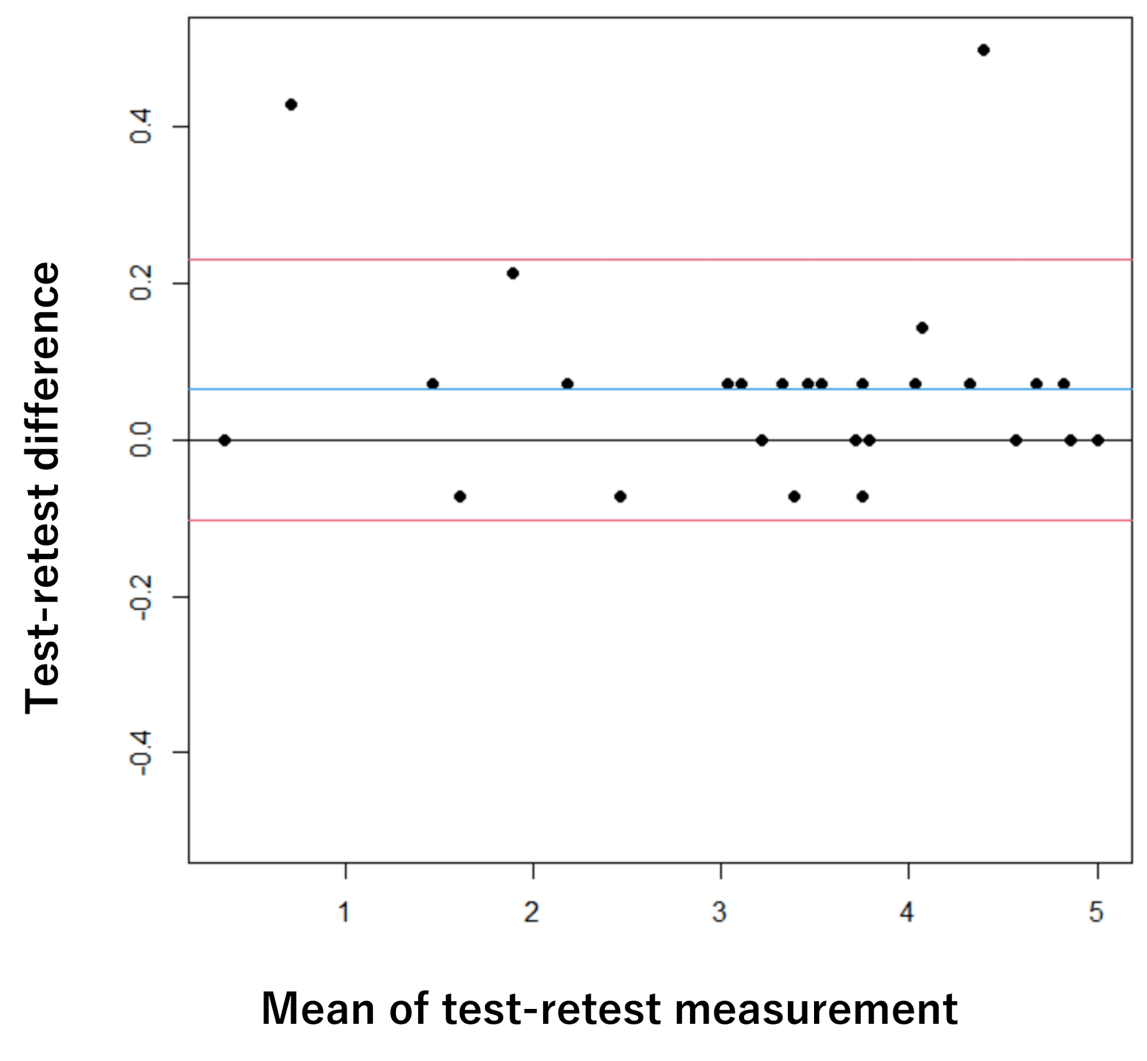
Bland-Altman plot describing the test-retest reliability of MAL(QOM) The blue line indicates the mean, the grey line indicates zero, and the red lines mark the lower and upper bounds of the 95% CI. MAL(QOM): Motor Activity Log (quality of movement), CI: confidence interval

The mean interval between the test and retest was 3.0 days (SD = 2.8), with a median of 3.0 days. Regarding test-retest reliability, the ICC (1,1) was 0.996 (95% CI: 0.991-0.998) for AOU and 0.994 (95% CI: 0.988-0.997) for QOM, showing extremely high values, with an SEM of 0.08 and an MDC of 0.23 for AOU and an SEM of 0.10 and an MDC of 0.28 for QOM (Table 4). In the Bland-Altman analysis, the mean difference for AOU was -0.05 points with an LOA of -0.20 to 0.09. For QOM, the mean difference was 0.06 points with an LOA of -0.10 to 0.23. Neither fixed bias nor proportional bias was observed (Figures 1-2).

**Table 4:** Test-retest and absolute reliability of MAL (n = 30) MAL(AOU): Motor Activity Log (amount of use), MAL(QOM): Motor Activity Log (quality of movement), SD: standard deviation, CI: confidence interval, LOA: limits of agreement, ICC: intraclass correlation coefficient, SEM: standard error of measurement, MDC: minimal detectable change

MAL	Test (SD)	Retest (SD)	ICC1.1 (95% CI)	SEM	MDC	LOA (95% CI for lower/upper LOA)
AOU	3.5 (1.3)	3.5 (1.3)	0.996 (0.991-0.998)	0.08	0.23	−0.2 to 0.09 (−0.24 to −0.16 / 0.05 to 0.13)
QOM	3.4 (1.3)	3.5 (1.3)	0.994 (0.988-0.997)	0.1	0.28	−0.1 to 0.23 (−0.15 to −0.05 / 0.18 to 0.28)

Validity

MAL(AOU) showed significant correlations with MAL(QOM) (r = 0.905, p < 0.05), FIM (r = 0.51, p < 0.05), VAS for pain during motion (r = -0.64, p < 0.05), affected grip strength (r = 0.50, p < 0.05), and QuickDASH (r = -0.87, p < 0.05). No significant correlation was found with unaffected grip strength (r = 0.29, p > 0.05).

Similarly, MAL(QOM) showed significant correlations with FIM (r = 0.43, p < 0.05), VAS for pain during motion (r = -0.51, p < 0.05), affected grip strength (r = 0.50, p < 0.05), and QuickDASH (r = -0.83, p < 0.05), with no significant correlation with unaffected grip strength (r = 0.32, p > 0.05) (Table 3).

## Discussion

To our knowledge, this study is the first to verify the psychometric properties of the Japanese version of the MAL in patients with upper extremity orthopedic disorders. The results showed that the AOU and QOM scales of the Japanese MAL possess sufficient internal consistency and extremely high intra-rater reliability (both relative and absolute). In terms of construct validity, the MAL showed strong correlations (convergent validity) with the QuickDASH and VAS, whereas it showed only moderate correlations (discriminant validity) with grip strength. These findings support the view that the MAL captures the independent construct of "performance" rather than merely serving as a surrogate marker for "capacity" (physical function). However, we acknowledge that correlations alone do not fully demonstrate an independent construct, and more detailed structural validity work is needed in the future to fully establish construct distinctiveness.

Reliability

Cronbach’s α for both AOU and QOM was 0.96, confirming high homogeneity and unidimensionality of the scale [12]. Regarding test-retest reliability, the ICC (1,1) was 0.99 or higher, indicating extremely high relative reliability [13]. A notable finding from the Bland-Altman analysis was the absence of fixed or proportional bias [16,17]. This implies that systematic measurement error is extremely small and that MAL score fluctuations are less susceptible to measurement error. Furthermore, the LOA width was extremely narrow (<0.5 points). Because this variation is substantially smaller than a single response category (1 point) on the 0-5 scale, it can be considered clinically trivial. In orthopedic disorders where daily conditions can fluctuate due to pain or limited ROM, ensuring absolute reliability to capture real change is a significant advantage for clinical application.

Validity

Construct validity was evaluated using a hypothesis-testing approach. First, the MAL showed a strong negative correlation (r = -0.87 to -0.83) with the QuickDASH. Since the QuickDASH evaluates "capacity/difficulty" of the upper extremity, the strong convergence with the "frequency/quality of use" measured by the MAL supports the notion that patients' subjective difficulty is closely linked to nonuse in daily life.

On the other hand, a clinically important finding was that the correlation between the MAL and affected grip strength remained moderate (r = 0.50). This indicates that the MAL does not measure the same construct as grip strength but captures a different aspect. Specifically, it suggests a discrepancy in which good physical capacity does not immediately translate into high performance in real life. Furthermore, the fact that the correlation between the MAL and VAS (r = -0.64) was stronger than that with grip strength suggests that, in orthopedic upper extremity disorders, "pain" may be a more decisive inhibiting factor for real-world use than muscle strength. This discrepancy between physical capacity and actual use aligns with our a priori hypothesis regarding the unique mechanisms of nonuse in this population. Unlike stroke patients, where "learned nonuse" typically originates from severe central motor and sensory deficits, our results imply that nonuse in orthopedic patients is closely linked to pain. This supports the model proposed by Punt et al. [7], which suggests that pain and subsequent fear-avoidance lead to a "protective nonuse." Importantly, this protective behavior can persist as a habit even when physical capacity, such as grip strength, is partially preserved or recovering. Therefore, the novelty of applying the MAL to orthopedic patients lies in its clinical utility for capturing this pain-driven behavioral adaptation, demonstrating that it measures a construct distinct from that measured by standard capacity tests. This supports the need for interventions to improve actual usage behavior in addition to tissue healing. These results statistically support the value of the MAL as a unique outcome measure independent of existing objective functional assessments.

Limitations

This study has some limitations. First, the sample size is small (n = 30) and insufficient for detailed structural validity examinations, such as factor analysis, as recommended by the COSMIN guidelines [19], warranting caution in generalizing the results. Second, although the target diseases included tendon and nerve disorders, our sample was predominantly composed of patients with fractures, which may limit the generalizability of our findings to other upper extremity orthopedic conditions. Future stratified analyses with larger samples are required to investigate disease-specific trends and the responsiveness of the MAL. Third, criterion validity was not fully established. Future studies should integrate wearable sensors, such as accelerometers, to objectively quantify real-world upper-limb activity and compare it with self-reported MAL scores, thereby strengthening criterion validity. Fourth, excluding patients with severe pain likely biased our sample toward less severe cases and constrained the range of VAS scores. Therefore, our findings may not be fully applicable to highly symptomatic acute or immediately post-operative patients, and further verification in these populations is needed. Fifth, we used convenience sampling for participant recruitment, which may have introduced selection bias and limited the generalizability of our findings to the broader population of patients with upper extremity orthopedic disorders. Sixth, although our sample size was sufficient to detect strong correlations, as determined by our a priori power analysis, it may be underpowered to detect more modest yet clinically meaningful associations (e.g., the weak correlations observed with unaffected-side grip strength, r = 0.29-0.32). Furthermore, a sample size of 30 is insufficient for robust psychometric evaluations, such as factor analysis. Seventh, we did not apply a multiple-testing correction when analyzing correlations, as we evaluated predetermined individual hypotheses for construct validity; however, this approach inherently increases the risk of type I errors.

## Conclusions

This study showed that the Japanese version of the MAL has high reliability and validity as a scale for evaluating the performance of the affected upper extremity in patients with orthopedic disorders. The MAL can measure the unique construct of "specific frequency and QOM in real life," which differs from conventional capacity tests and subjective difficulty measures. Clinically, using the MAL makes it possible to quantitatively capture discrepancies where functional improvement does not directly translate into real-world use, as well as protective nonuse due to pain. Given that the ultimate goal of upper extremity orthopedic rehabilitation is not only functional recovery but also adaptation to daily life and social participation, we recommend developing a comprehensive evaluation system that integrates the MAL with existing measures, such as the QuickDASH. While the QuickDASH primarily captures subjective difficulty and symptoms (capacity), the MAL uniquely assesses actual performance. Integrating these tools allows clinicians to identify discrepancies between what a patient can do and what they actually do, leading to a more targeted and effective rehabilitation strategy.
